# Predicting new-onset stroke with machine learning: development of a model integrating traditional Chinese and western medicine

**DOI:** 10.3389/fphar.2025.1546878

**Published:** 2025-02-21

**Authors:** Liuding Wang, Jingzi Shi, Lina Miao, Yifan Chen, Jingjing Wei, Min Jia, Zhiyi Gong, Ze Yang, Jian Lyu, Yunling Zhang, Xiao Liang

**Affiliations:** ^1^ Departmalet of Neurology, Xiyuan Hospital, China Academy of Chinese Medical Sciences, Beijing, China; ^2^ Graduate School, Beijing University of Chinese Medicine, Beijing, China; ^3^ Departmalet of Cardiology, Xiyuan Hospital, China Academy of Chinese Medical Sciences, Beijing, China; ^4^ Medical Ethics Committee, Xiyuan Hospital, China Academy of Chinese Medical Sciences, Beijing, China; ^5^ Shandong University of Traditional Chinese Medicine, Jinan, China; ^6^ NMPA Key Laboratory for Clinical Research and Evaluation of Traditional Chinese Medicine, Xiyuan Hospital, China Academy of Chinese Medical Sciences, Beijing, China; ^7^ National Clinical Research Center for Chinese Medicine Cardiology, Xiyuan Hospital, China Academy of Chinese Medical Sciences, Beijing, China

**Keywords:** artificial intelligence, combination of disease and syndrome, prevention strategy, populations at high risk of stroke, traditional medicine

## Abstract

**Introduction:**

The integration of traditional Chinese medicine (TCM) and Western medicine has demonstrated effectiveness in the primary prevention of stroke. Therefore, our study aims to utilize TCM syndromes alongside conventional risk factors as predictive variables to construct a machine learning model for assessing the risk of new-onset stroke.

**Methods:**

We conducted a ten-year follow-up study encompassing 4,511 participants from multiple Chinese community hospitals. The dependent variable was the occurrence of the new-onset stroke, while independent variables included age, gender, systolic blood pressure (SBP), diabetes, blood lipids, carotid atherosclerosis, smoking status, and TCM syndromes. We developed the models using XGBoost in conjunction with SHapley Additive exPlanations (SHAP) for interpretability, and logistic regression with a nomogram for clinical application.

**Results:**

A total of 1,783 individuals were included (1,248 in the training set and 535 in the validation set), with 110 patients diagnosed with new-onset stroke. The logistic model demonstrated an AUC of 0.746 (95% *CI*: 0.719–0.774) in the training set and 0.658 (95% *CI*: 0.572–0.745) in the validation set. The XGBoost model achieved a training set AUC of 0.811 (95% *CI*: 0.788–0.834) and a validation set AUC of 0.628 (95% *CI*: 0.537–0.719). SHAP analysis showed that elevated SBP, Fire syndrome in TCM, and carotid atherosclerosis were the three most important features for predicting the new-onset stroke.

**Conclusion:**

Under identical traditional risk factors, Chinese residents with Fire syndrome may have a higher risk of new-onset stroke. In high-risk populations for stroke, it is recommended to prioritize the screening and management of hypertension, Fire syndrome, and carotid atherosclerosis. However, future high-performance TCM predictive models require more objective and larger datasets for optimization.

## 1 Introduction

Stroke presents a significant global public health challenge, characterized by high incidence rates, substantial disability, and mortality (Virani et al., 2020). The lifetime risk of stroke among adults aged 25 and older is estimated to be as high as 25% (Feigin et al., 2018). In China, over 2.4 million new stroke cases are recorded annually, resulting in approximately 1.1 million stroke-related deaths (Wang et al., 2017). Projections indicate that the global burden of stroke will continue to escalate in the coming decade (Ouriques Martins et al., 2019; Wu et al., 2019; King et al., 2020). Despite extensive efforts over the past decades to mitigate stroke incidence through the improved management of hypertension, diabetes, and dyslipidemia, outcomes have remained unsatisfactory. Recently, traditional Chinese medicine (TCM) has gained prominence as a preventive measure for primary stroke intervention. Nevertheless, standardized and effective TCM strategies for populations at high risk of stroke have yet to be firmly established. Guiding TCM practitioners to accurately prevent first strokes remains a considerable challenge.

The integration of disease and syndrome concepts represents a predominant model in the research of integrated Chinese and Western medicine, synthesizing the strengths of modern medicine’s “disease differentiation” with TCM’s “syndrome differentiation.” This methodology is deemed crucial for the standardization and enhancement of TCM practices within the contemporary medical landscape. This study aims to develop a predictive model for new-onset stroke risk based on the combined frameworks of disease and syndrome, thus providing a tool for identifying individuals who may benefit from TCM in stroke prevention. In this context, “disease” pertains to individuals at high risk of stroke prior to its onset, while “syndrome” refers to symptom clusters categorized according to TCM theory.

## 2 Materials and methods

### 2.1 Study population

This study was conducted as part of the Stroke Screening and Prevention Project under the National Health Commission’s Major Special Project on Healthcare Reform. In collaboration with 12 community hospitals in Beijing, our research team screened 56,389 individuals for stroke risk. From this cohort, comprehensive data were gathered for 12,654 individuals identified as high-risk for stroke, forming the basis of a study database. A subset of 5,999 participants from this database, recruited between June 2012 and February 2013 from Wangzuo Town, Puhuangyu Community, Huaxiang Community, and Fangzhuang Community in Fengtai District, Beijing, was selected for remote follow-up over 10 years to track stroke incidence. All participants signed informed consent forms upon enrollment. The study received ethical approval from the Ethics Committee of Xiyuan Hospital, China Academy of Chinese Medical Sciences (Approval No.: 2022XLA117-1). The study was registered on Chinese Clinical Trial Registry (ChiCTR2200063905).

High-risk individuals for stroke were defined as those with three or more risk factors, or with a history of stroke or transient ischemic attack. Risk factors included hypertension (blood pressure ≥140/90 mmHg or antihypertensive medication use), dyslipidemia, diabetes mellitus, atrial fibrillation, valvular heart disease, smoking history, significant overweight or obesity, lack of physical activity, and a family history of stroke. Stroke diagnoses adhered to the 2018 Chinese Guidelines for Acute Ischemic Stroke (Chinese Society of Neurology and Chinese Stroke Society, 2018) and the 2019 Chinese Guidelines for Cerebral Hemorrhage (Chinese Society of Neurology and Chinese Stroke Society, 2019). Inclusion criteria encompassed individuals at high risk of stroke with complete baseline data; exclusion criteria ruled out those with a prior stroke history and individuals with psychiatric, cognitive, or emotional disorders.

**FIGURE 1:**
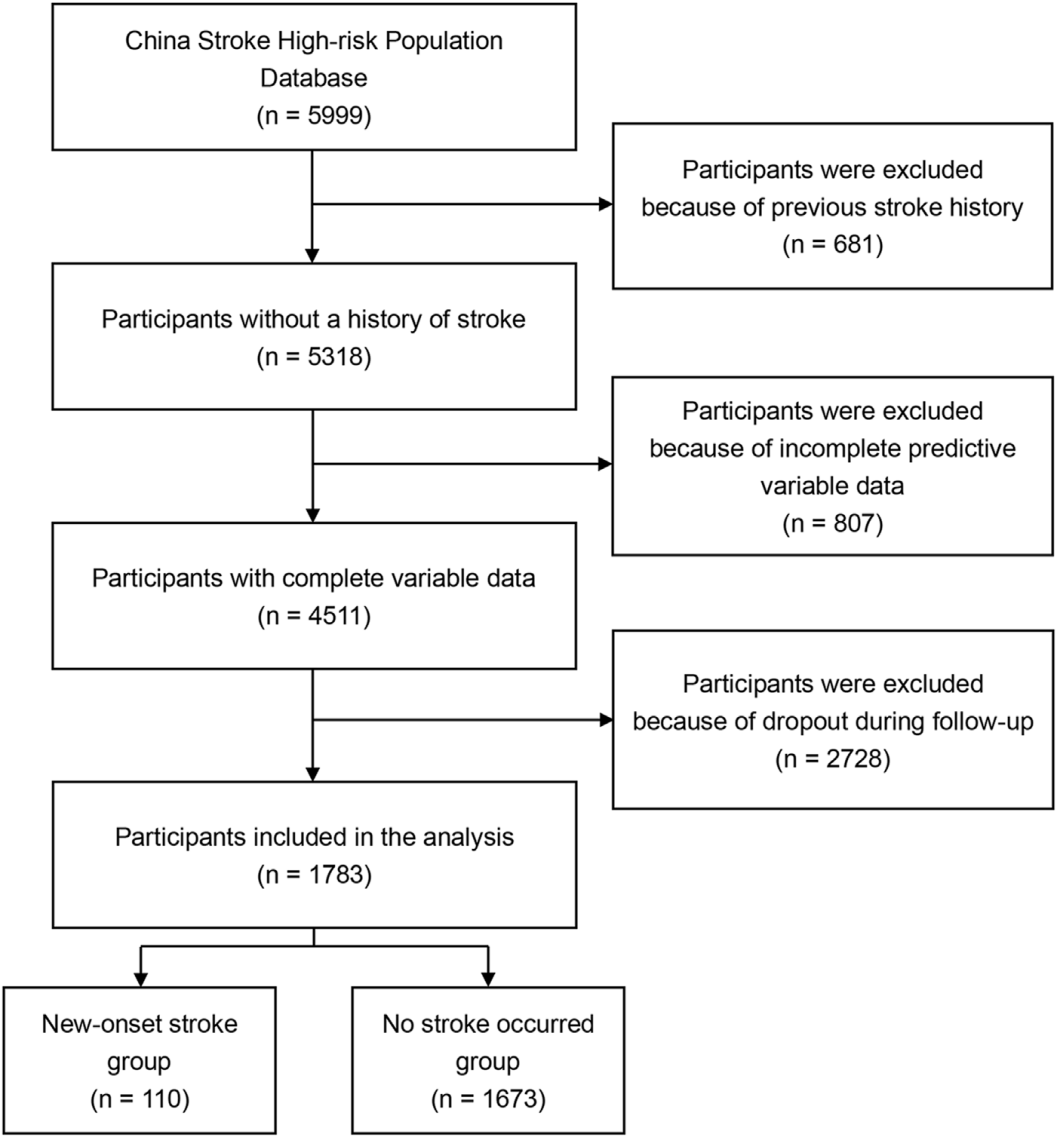
Flowchart of the participants selection process.

Following eligibility assessment, we initially excluded 681 individuals due to pre-existing stroke at enrollment. Additionally, 807 participants were excluded after confirming the completeness of key variables such as systolic blood pressure (SBP) and carotid ultrasound results. During follow-up, another 2,728 participants were excluded due to loss to follow-up or lack of cooperation, making it impossible to ascertain stroke incidence over the decade. Consequently, the final cohort for model development and validation included 1,783 participants (Figure 1).

### 2.2 Data collection

We collected the following data: demographic information, lifestyle factors, family and cardiovascular histories, physical examination, laboratory tests, and carotid ultrasound results using a standardized questionnaire. Additionally, TCM symptoms were documented using a specialized TCM questionnaire by trained practitioners.

### 2.3 Predictive variables extraction

According to existing stroke risk models, literature reviews, and expert consultations, we identified candidate predictors. Modern medicine predictors included: age, gender, SBP levels, antihypertensive treatment, diabetes mellitus, total cholesterol (TC), total triglycerides (TG), low-density lipoprotein cholesterol (LDL-C), high-density lipoprotein cholesterol (HDL-C), carotid atherosclerosis, and current smoking status. TCM predictors included six syndromes: Qi deficiency, Fire, Yang deficiency, Yin deficiency, Phlegm-dampness, and Blood stasis, each classified as binary variables.

Two researchers independently extracted predictor information from the database and cross-verified their findings, resolving any discrepancies through discussion.

### 2.4 Sample size calculation

Sample size calculation was conducted using the Events Per Variable (EPV) method (Peduzzi et al., 1996). Ideally, the EPV should exceed ten, meaning the number of participants with clinical outcomes should be at least ten times the number of independent variables. This study aims to incorporate ten predictors, necessitating a minimum of 100 cases of first stroke events.

### 2.5 Handling imbalanced samples

In R version 3.6.2, we balanced the positive and negative samples using random over-sampling (ROS), random under-sampling (RUS), and synthetic data generation (SDG) methods. ROS randomly replicates positive samples to achieve a 1:1 ratio of positive to negative samples. This may lead to model overfitting due to increased duplicate observations. RUS randomly reduces negative samples to achieve the same 1:1 ratio. This results in significant loss of sample information and potential inaccuracies in the results. The SDG method used in our study employs a smoothed bootstrap approach. It generates synthetic balanced samples that closely mirror the original data’s characteristics (Blagus and Lusa, 2010). Compared to the other two methods, the SDG method might better represent the true characteristics of the samples and demonstrate the effectiveness of the models.

### 2.6 Statistical analysis

For continuous data, we used the Kolmogorov-Smirnov test to check for normality. Normally distributed data are reported as “mean ± standard deviation.” We compared groups using the t-test. Skewed data are reported as “median (interquartile range).” We compared groups using the Mann-Whitney U test. Categorical variables are reported as “frequency (percentage).” We compared groups using the chi-square test.

We randomly divided the participants into a training set (1248 participants, 70%) and a validation set (535 participants, 30%). Candidate predictors were preliminarily screened using bidirectional stepwise regression based on the Akaike Information Criterion (AIC) and Bayesian Information Criterion (BIC). We performed a collinearity test to check for multicollinearity among predictors. Predictors were selected based on both statistical and clinical significance. Subsequently, we developed prediction models using logistic regression and XGBoost machine learning. After model construction, both the logistic and XGBoost models were validated internally. Model performance was assessed using the area under the curve (AUC) for discrimination and the Brier score for calibration. AUC values range from 0.5 to 1, with higher values indicating better discrimination. Brier scores range from 0 to 1, with lower values indicating better performance. Calibration was evaluated using calibration curves, which compare predicted and actual probabilities. Decision curve analysis was used to assess the practical value of the models. We created a nomogram to visualize the logistic model. SHapley Additive exPlanations (SHAP) were employed to interpret the XGBoost model. All statistical analyses were performed using R 3.6.2.

## 3 Results

### 3.1 Baseline characteristics of participants

A total of 1783 individuals at high risk of stroke were included, consisting of 110 first stroke cases and 1673 non-stroke controls, with an overall male proportion of 35.67%. In the stroke group, males constituted 46.36%, with median age, SBP, and HDL-C levels of 62 (14) years, 143.5 (25) mmHg, and 1.12 (0.38) mmol/L, respectively. In the non-stroke group, males accounted for 34.97%, with median age, SBP, and HDL-C levels of 59 (12) years, 135 (25) mmHg, and 1.20 (0.43) mmol/L, respectively. Among stroke participants, 40.00% had diabetes, 90.91% had carotid atherosclerosis, and 29.09% were smokers, compared to 29.29%, 71.19%, and 20.20%, respectively, in the non-stroke group. Regarding TCM characteristics, the Fire syndrome was the most prevalent in both groups, observed in 67.27% of stroke patients and 50.27% of non-stroke individuals. Significant differences between groups were noted for SBP, HDL-C, carotid atherosclerosis, and the Fire syndrome (*P* ≤ 0.001), while no significant differences were observed for TC (*P* = 0.951), TG (*P* = 0.257), or LDL-C (*P* = 0.853). Single factor analysis is summarized in Table 1. The dataset exhibited a severe imbalance between stroke and non-stroke cases (approximately 15:1), prompting the use of ROS, RUS, and SDG methods to balance the samples (Supplementary Table S1).

**TABLE 1:** Baseline characteristics of the new-onset stroke group and no stroke occurred group.

Variables	New-onset stroke (n = 110)	No stroke occurred (n = 1673)	*χ2/Z*	*P*
Male, n (%)	51 (46.36)	585 (34.97)	5.842	0.016
Age, years	62 (14)	59 (12)	2.823	0.005
Systolic blood pressure, mmHg	143.5 (25)	135 (25)	4.303	<0.001
Diabetes, n (%)	44 (40.00)	490 (29.29)	5.644	0.018
Total cholesterol, mmol/L	4.98 ± 1.01	5.09 ± 1.02	-	0.951
Triglyceride, mmol/L	1.70 (1.13)	1.57 (1.06)	1.134	0.257
LDL-C, mmol/L	3.23 (1.25)	3.21 (1.18)	0.186	0.853
HDL-C, mmol/L	1.12 (0.38)	1.2 (0.43)	−3.183	0.001
Carotid atherosclerosis, n (%)	100 (90.91)	1191 (71.19)	20.088	<0.001
Current smoking, n (%)	32 (29.09)	338 (20.20)	4.958	0.026
Fire syndrome, n (%)	74 (67.27)	841 (50.27)	11.945	0.001
Yang deficiency syndrome, n (%)	29 (26.36)	590 (35.27)	3.609	0.057
Blood stasis syndrome, n (%)	2 (1.82)	153 (9.15)	6.981	0.008
Yin deficiency syndrome, n (%)	37 (33.64)	551 (32.93)	0.023	0.880
Qi deficiency syndrome, n (%)	60 (54.55)	809 (48.36)	1.583	0.208
Phlegm-dampness syndrome, n (%)	14 (12.73)	330 (19.73)	3.246	0.072

Data are presented as absolute number (percentage), mean ± standard deviation, or median (interquartile range). HDL-C, high-density lipoprotein cholesterol; LDL-C, low-density lipoprotein cholesterol.

### 3.2 Screening of predictive variables

Based on the SDG-generated sample data, bidirectional stepwise regression guided by the AIC and BIC was employed for variable screening (Supplementary Tables S2, S3). A collinearity test revealed significant collinearity among Fire, Qi deficiency, Yang deficiency, and Yin deficiency (tolerance <0.2, variance inflation factor >5) (Supplementary Table S4). The strong collinearity between Fire and other factors explains its exclusion from the bidirectional stepwise regression, despite a statistically significant difference between groups. Clinically, bidirectional stepwise regression suggested that Yang deficiency, Phlegm-dampness, and Blood stasis act as protective factors, contrary to clinical expectations. Clinical experts indicated that Fire is closely associated with the pathogenesis of stroke and is likely a significant risk factor.

Considering the results of the bidirectional stepwise regression, collinearity, and clinical interpretability, eight variables were included in the final model: gender, age, SBP, diabetes, HDL-C, carotid atherosclerosis, current smoking, and Fire.

### 3.3 Development and validation of the prediction model

**FIGURE 2:**
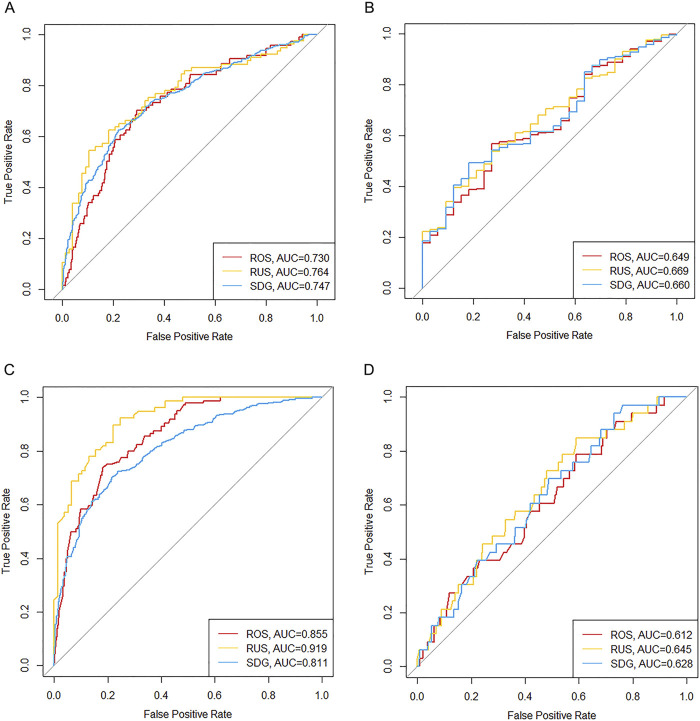
Receiver operating characteristic curves of predictive models in training and validation sets. **(A)** Logistic regression models in the training set. **(B)** Logistic regression models in the validation set. **(C)** XGBoost models in the training set. **(D)** XGBoost models in the validation set. AUC, area under the curve; ROS, random over-sampling; RUS, random under-sampling; SDG, synthetic data generation.

**FIGURE 3:**
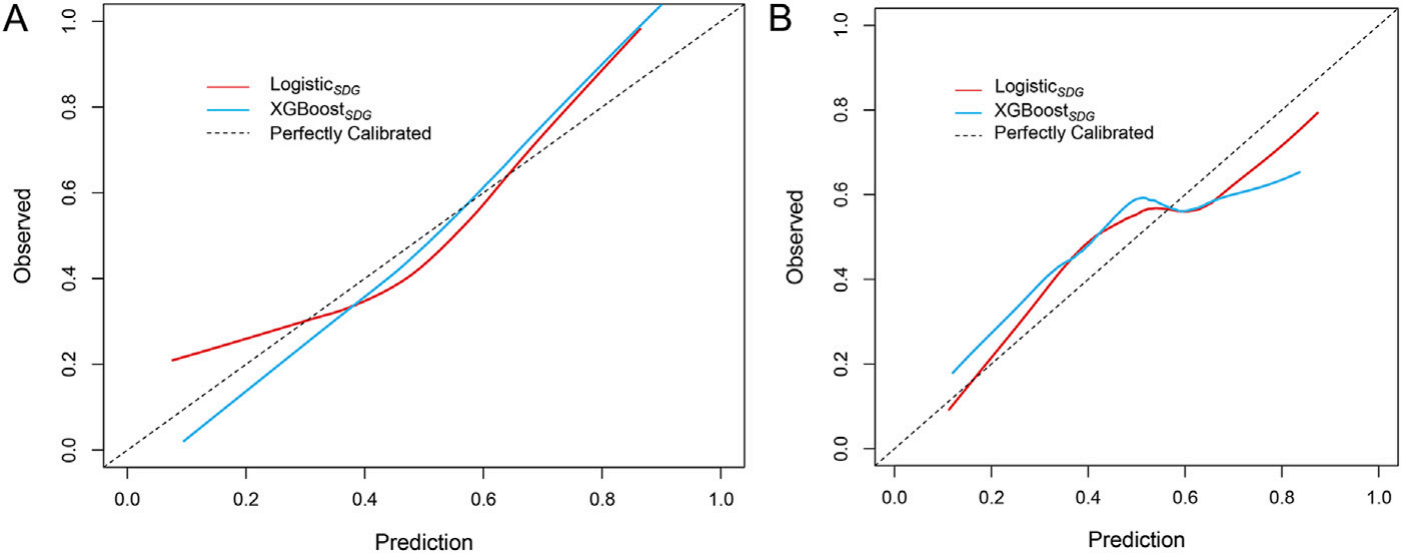
Calibration curves of predictive models in the training set **(A)** and validation set **(B)**.

**FIGURE 4:**
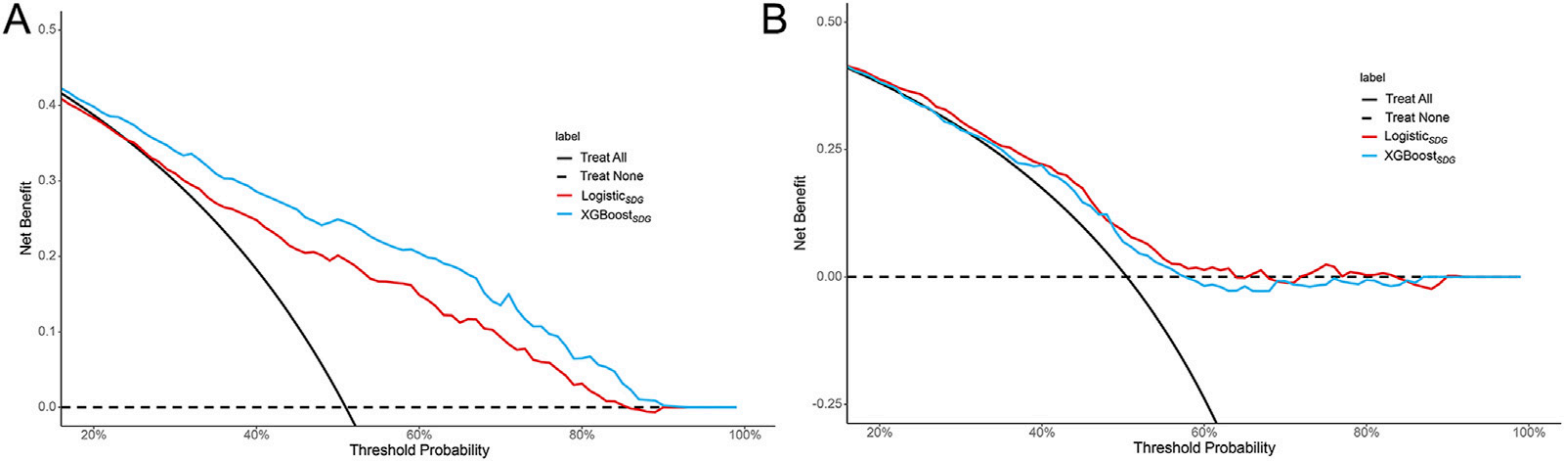
Decision curve analyses of predictive models in the training set **(A)** and validation set **(B)**.

Using the logistic_*SDG*_ model as an example, regression coefficients are presented in Table 2. The model achieved an AUC of 0.746 and a Brier score of 0.207 in the training set, while in the validation set, the AUC was 0.658 with a Brier score of 0.219. These results indicate the model possesses predictive capabilities. The performance of logistic and XGBoost models is summarized in Table 3 and illustrated in Figure 2. We compared the performance of the logisticSDG model with that of the XGBoost_*SDG*_ model. DeLong’s test indicated superior discrimination for the XGBoost_*ROS*_ model in the training set (*P* < 0.001); however, in the validation set, there was no statistical difference in discrimination between the two models. We further assessed the applicability of the models using calibration curves (Figure 3) and decision curves (Figure 4). The results showed that the predicted probabilities were well-balanced with the actual probabilities and the models possessed practical clinical utility within certain threshold ranges.

**TABLE 2:** Logistic regression stroke risk prediction model based on synthetic data generation samples.

Features	Regression coefficient	OR	*95%* CI	*P*
(Intercept)	−2.36			<0.001
Sex	0.190	1.21	0.90–1.62	0.206
Age	0.0214	1.02	1.01–1.03	<0.001
SBP	0.0192	1.02	1.01–1.03	<0.001
Diabetes	0.286	1.33	1.02–1.73	0.033
HDL-C	−0.549	0.58	0.41–0.82	0.002
Carotid atherosclerosis	1.13	3.09	2.11–4.58	<0.001
Current smoking	0.604	1.83	1.29–2.60	<0.001
Fire syndrome	0.657	1.93	1.50–2.49	<0.001

CI, confidence interval; HDL-C, high-density lipoprotein cholesterol; OR, odds ratio; SBP, systolic blood pressure.

**TABLE 3:** Predictive efficiency of logistic regression and XGBoost models for stroke risk prediction.

	AUC	95% CI	Cut-off	Sensitivity	Specificity	Brier score
Training set						
Logistic_*ROS*_	0.73	0.71–0.750	0.538	0.701	0.708	0.212
Logistic_*RUS*_	0.764	0.688–0.841	0.571	0.623	0.818	0.198
Logistic_*SDG*_	0.746	0.719–0.774	0.582	0.623	0.781	0.207
XGBoost_*ROS*_	0.855	0.840–0.870	0.552	0.738	0.817	0.165
XGBoost_*RUS*_	0.919	0.878–0.959	0.382	0.922	0.753	0.131
XGBoost_*SDG*_	0.811	0.788–0.834	0.521	0.703	0.784	0.180
Validation set						
Logistic_*ROS*_	0.649	0.559–0.74	0.463	0.568	0.727	0.219
Logistic_*RUS*_	0.667	0.582–0.752	0.395	0.476	0.788	0.227
Logistic_*SDG*_	0.658	0.572–0.745	0.429	0.49	0.818	0.219
XGBoost_*ROS*_	0.612	0.516–0.708	0.317	0.788	0.410	0.189
XGBoost_*RUS*_	0.645	0.554–0.737	0.328	0.848	0.410	0.249
XGBoost_*SDG*_	0.628	0.537–0.719	0.411	0.697	0.512	0.202

AUC, area under the curve; CI, confidence interval; ROS, random over-sampling; RUS, random under-sampling; SDG, synthetic data generation.

### 3.4 Logistic model visualization

**FIGURE 5:**
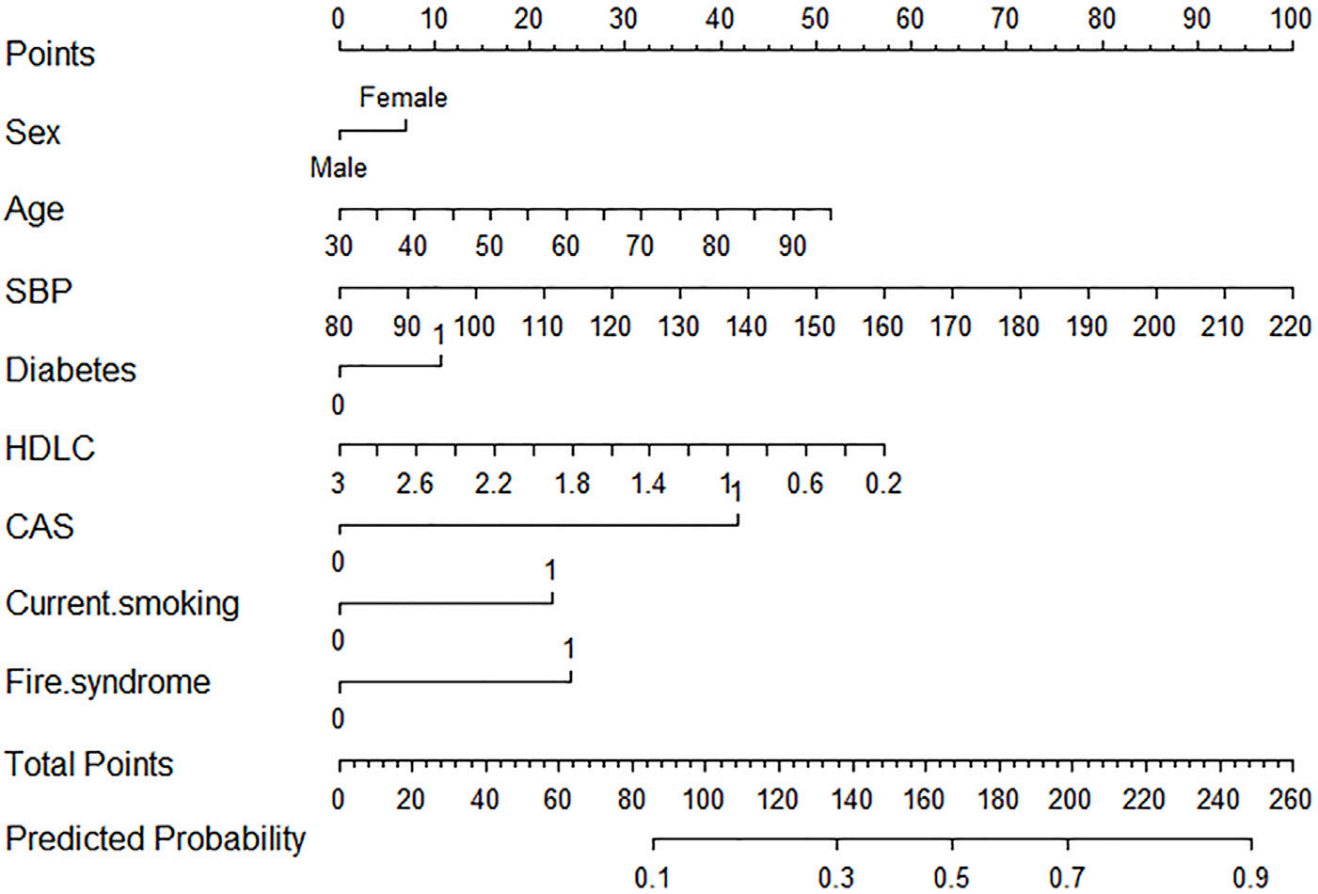
Nomogram of logistic regression model for stroke risk prediction using synthetic data generation samples. CAS, carotid atherosclerosis; HDL-C, high-density lipoprotein cholesterol; SBP, systolic blood pressure.

A nomogram illustrating the logistic_*SDG*_ model is presented in Figure 5. Age, SBP, and HDL-C are treated as continuous variables, while the remaining variables are categorical. Based on a Youden index of 0.40, the optimal cutoff value on the nomogram was determined to be 180, yielding high sensitivity (62.3%) and specificity (78.1%). Scores above 180 suggest a high risk of first stroke occurrence.

### 3.5 XGBoost model explanation

**FIGURE 6:**
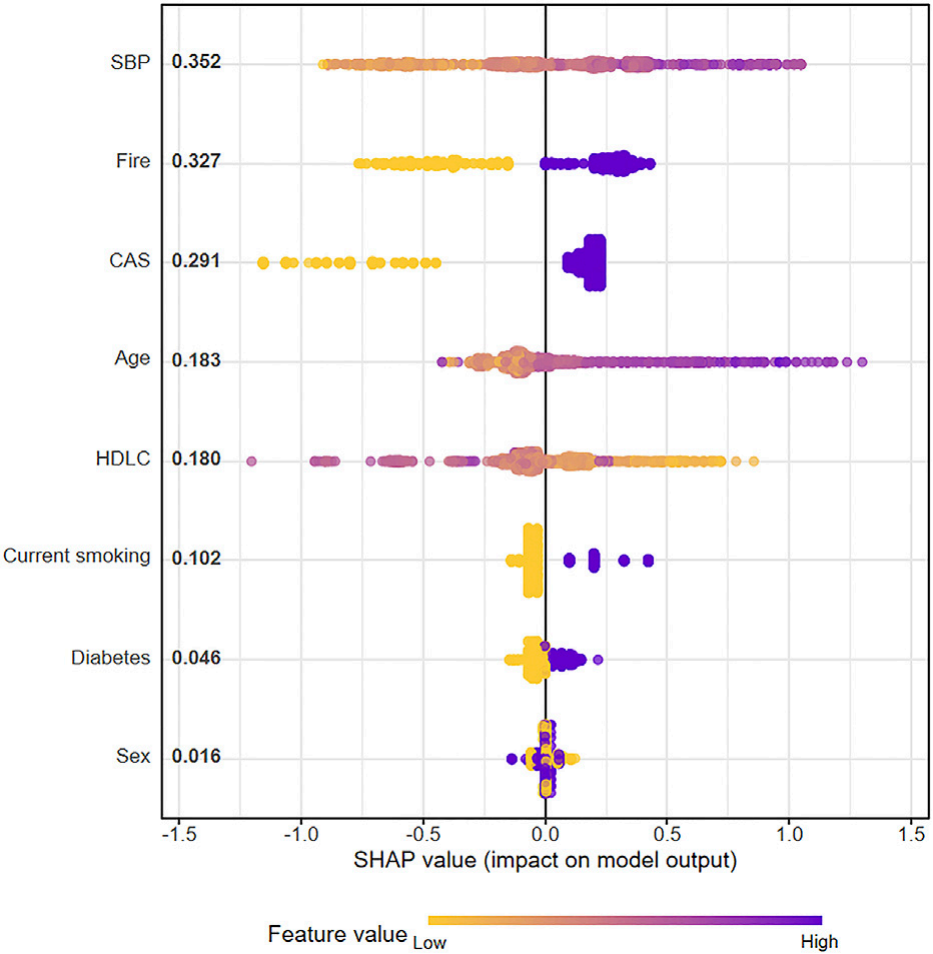
SHAP plot of XGBoost model for stroke risk prediction using synthetic data generation samples.

We used SHAP to visually explain how each included variable influences the stroke prediction of the XGBoost_*SDG*_ model. Figure 6 illustrates the eight features included in the model, with each feature’s significance line indicating its contribution to the prediction outcome. The color variation represents changes in feature values, where purple indicates high values and yellow indicates low values. The distance of a point from the baseline SHAP value of zero reflects its impact on the output. For example, higher SBP and older age, shown in the extended right tail, are strongly linked to increased stroke risk. In contrast, higher HDL-C levels in the extended left tail show a protective effect against stroke.

**FIGURE 7:**
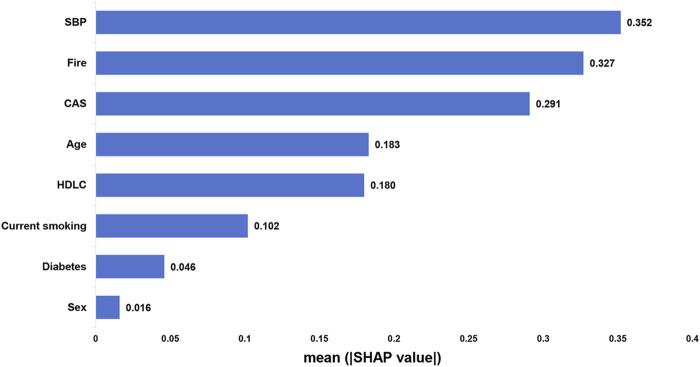
Bar chart displaying variable contributions based on the absolute values of SHAP.

Figure 7 shows the ranking of eight features based on their average absolute SHAP values, with the SHAP values on the x-axis indicating the importance of each feature in the prediction model. Elevated SBP has the most significant impact on stroke risk, followed by the Fire syndrome. Notably, the impact of Fire syndrome on stroke risk is greater than that of traditional cerebrovascular risk factors such as diabetes and current smoking.

**FIGURE 8:**
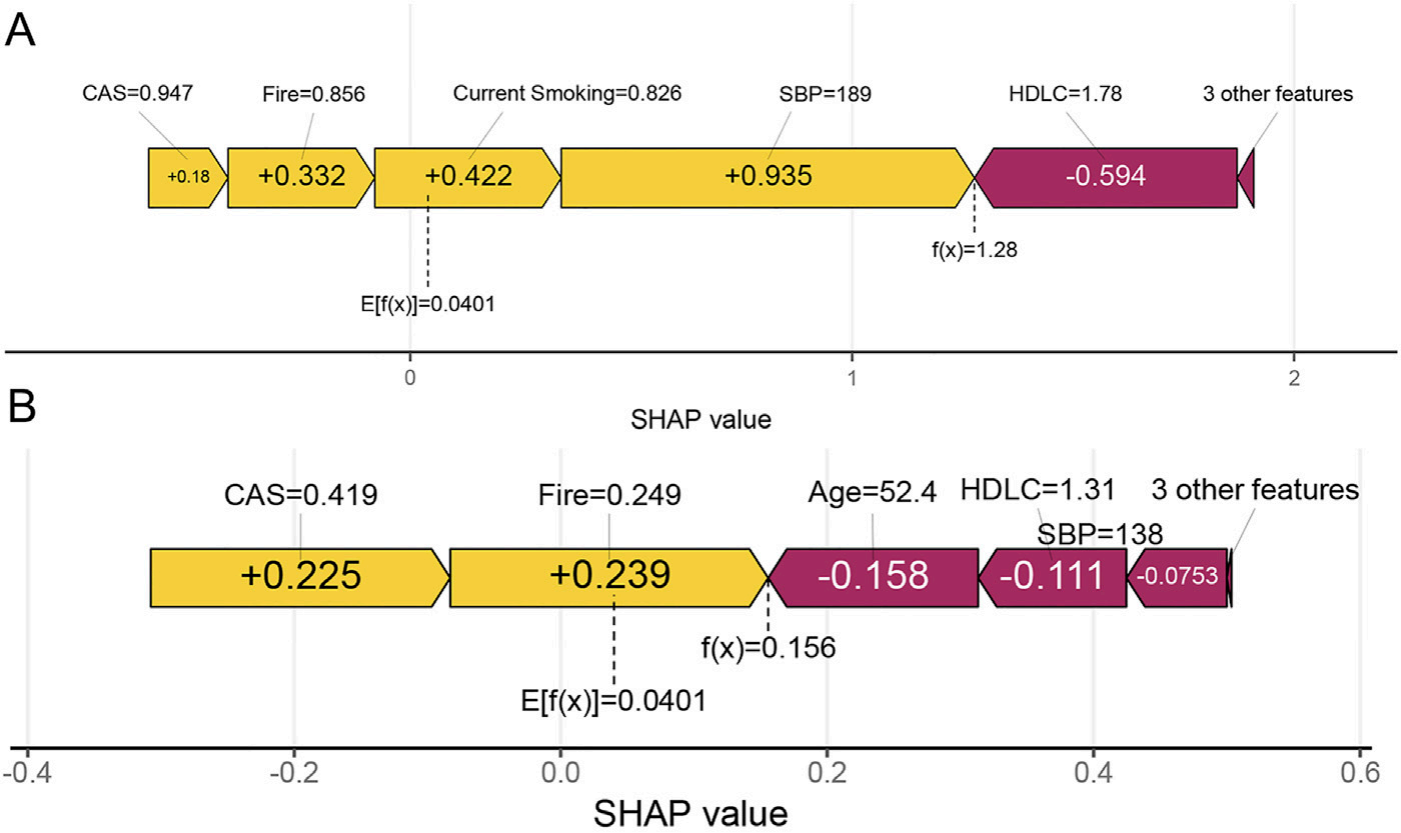
Individual prediction of stroke risk. **(A)** An individual who experienced a stroke. **(B)** An individual without stroke. The f(x) represents the model’s output, which is the predicted probability for the selected individual. Features that increase the prediction are shown in yellow, while features that decrease the prediction are shown in purple. Yellow features are represented by right arrows, and purple features are represented by left arrows.

Additionally, the interpretability of the model is demonstrated through two randomly selected patient examples. One example involves an individual who experienced a stroke, with a high SHAP prediction score of 1.28 (Figure 8A), whereas the other example is of an individual who did not have a stroke, with a lower SHAP prediction score of 0.156 (Figure 8B).

## 4 Discussion

### 4.1 Summary of findings

Our study used baseline data from 1,783 high-risk individuals for stroke in Chinese communities and information on whether they developed a stroke during a ten-year follow-up period. Logistic regression and XGBoost methods were applied to develop a predictive model for first-ever stroke that combines traditional Chinese and Western medicine features. The results showed that the top risk factors for first-ever stroke, ranked by importance, were high SBP, Fire syndrome, carotid atherosclerosis, advanced age, current smoking, and diabetes, while HDL-C acted as a protective factor. Both the logistic and XGBoost models showed similar performance, with good discrimination in the training set but slightly reduced discrimination in the validation set. Both models showed satisfactory calibration, and the decision curves indicated that they have practical clinical utility within certain threshold ranges. Our study carries the following clinical and research implications: First, it identifies Fire syndrome as an important risk factor for predicting first-ever stroke, which is crucial for combined TCM-Western medicine primary prevention of stroke. Greater attention should be paid to the identification and management of Fire syndrome in future clinical practice. Second, it demonstrates the feasibility of building predictive models by combining TCM syndromes with modern medical risk factors. This offers a strong example of developing an integrated TCM and Western medicine prevention model for other major diseases.

### 4.2 Comparison with existing models

We compared our study with established stroke risk prediction models. The Framingham Stroke Profile (FSP) is widely used to estimate ten-year stroke probability by incorporating risk factors such as age, SBP, antihypertensive treatment, diabetes, smoking, cardiovascular disease, atrial fibrillation, and left ventricular hypertrophy (Wolf et al., 1991). The modified FSP model, when applied to Chinese populations, has been shown to overestimate stroke incidence (Huang et al., 2013). For instance, among Chinese individuals with a modified FSP score of 10–12, the actual stroke incidence is much lower than the predicted rate. Additionally, the AUC for predicting stroke in Chinese men is 0.726, and for women, it is 0.656, indicating poor performance. The model’s limitations in discrimination and calibration may be due to differences in population characteristics. However, the predictive variables used in this model form a crucial foundation for subsequent models. In our study, we also included age, SBP, diabetes, and current smoking, similar to the FSP, to ensure basic predictive ability.

To address the shortcomings of the modified FSP in predicting stroke risk in the Chinese population, experts developed an atherosclerotic cardiovascular disease risk prediction model based on Chinese population data. This model similarly includes traditional risk factors such as gender, age, HDL-C levels, and blood pressure (Xing et al., 2019). Although the model has been validated in the Chinese population, its effectiveness in accurately identifying high-risk individuals for stroke remains limited (Zhang Y. et al., 2020). It’s worth noting that despite being developed in 2019, the original data used to build the model were collected in 1998. To improve the predictive performance of the model, using more recent population data and incorporating new features are both important. Our study used data collected between 2012 and 2013. Additionally, our study innovatively included TCM syndromes as important features.

Our study combines TCM syndromes with the traditional risk factors mentioned above, distinguishing it from previous research. TCM syndromes reflect the overall state of the body, contrasting with measures such as blood pressure or cholesterol, which, although precise, focus on specific aspects rather than the whole body. Stroke is frequently associated with changes in the overall state of the body. Therefore, by considering this characteristic of TCM syndromes, we included them to enhance the model’s predictive ability. Specifically, our findings suggest that Fire syndrome may be a critical factor in identifying individuals at a higher risk of stroke, potentially complementing traditional risk models by capturing additional pathophysiological dimensions.

### 4.3 The role of TCM syndromes in stroke prediction

The SHAP analysis demonstrated that Fire syndrome in TCM is the second most important risk factor for first stroke, following hypertension. This suggests that screening and managing Fire syndrome should be emphasized in clinical practice. The symptoms associated with Fire include halitosis, a bitter taste in the mouth, a sticky sensation in the mouth, excessive thirst, a red tongue with yellow coating, insomnia, and dreaminess. Managing Fire syndrome involves methods such as taking Fire-clearing Chinese herbs, practicing the Qigong (Baduanjin and Tai Chi), and maintaining a pleasant mood. Why Fire causes stroke can be explained using TCM theory. Fire is characterized as a Yang pathogen with proactive qualities, often leading to disease onset and progression. During the complex pathological transition from a pre-stroke state to the occurrence of stroke, Fire plays a crucial role in facilitating these pathological transformations. Specifically, it promotes the conversion of pathological products such as phlegm and blood stasis into toxins (Tanyuhuadu) (Liu et al., 2024). Renowned TCM scholars, including Liu Wansu of the Jin Dynasty, Zhu Danxi of the Yuan Dynasty, and Ye Tiansi of the Qing Dynasty, have all suggested that Fire can lead to stroke. TCM posits that the sources of Fire primarily include the consumption of large quantities of high-fat and high-calorie foods, as well as prolonged negative emotional states. A clinical study has shown that the herbal formula Danggui Liuhuang Tang, known for its effects of clearing Fire, can reduce the release of proinflammatory cytokines and vascular endothelial growth factor by inhibiting the JAK2/STAT3 signaling pathway, thereby improving vascular endothelial function in type 2 diabetes mellitus patients with Fire syndrome (Xu et al., 2024). An animal experiment has demonstrated that Huanglian Jiedu Tang, a clearing Fire herbal formula, can reduce oxidative stress and atherosclerotic changes in ApoE-deficient mice on a high-fat diet (Yang et al., 2024). This is achieved by regulating lipid metabolism and enhancing arginine biosynthesis. Additionally, geniposide, a clearing Fire herb with homology of medicine and food, can increase CXCL14 expression in perivascular adipose tissue and induces M2 polarization of plaque macrophages, slowing atherosclerosis progression in mice (He et al., 2023). These findings indicate that the biological mechanisms linking the TCM Fire syndrome to stroke risk might involve abnormal lipid metabolism, excessive release of proinflammatory and vascular endothelial factors, and oxidative stress. Therefore, clearing Fire may reduce stroke risk by improving these pathological processes.

### 4.4 The role of traditional vascular risk factors in stroke prediction

Regarding the impact of blood pressure for stroke risk, SBP and antihypertensive treatment were identified as key hypertension-related predictors in our model. Hypertension is a primary modifiable risk factor for stroke. In elderly populations, SBP has a stronger correlation with stroke risk compared to diastolic blood pressure (Sethi et al., 2021). A longer duration of hypertension also increases the risk of ischemic stroke, highlighting its significance in risk prediction (Kim et al., 2019). Strict control of SBP throughout periods of hypertension can help mitigate this risk. However, a study conducted in the UK found that prolonged hypertension duration elevates cardiovascular risk regardless of blood pressure management (Zheng et al., 2022). Consequently, our model, which utilizes a single SBP measurement (0.0192*SBP), may overlook the cumulative effects of hypertension. Cumulative blood pressure, which considers both exposure duration and levels, provides a more comprehensive view of long-term blood pressure trends and offers superior predictive value compared to isolated SBP measurements (Pool et al., 2018; Liu et al., 2021; Nwabuo et al., 2021; Wang et al., 2022). Additionally, we included antihypertensive treatment to model its interaction with SBP, akin to the NEWHRXSBP variable in the modified FSP. However, logistic regression revealed minimal correlation between this variable and stroke incidence, resulting in its exclusion from the final model.

Diabetes is recognized as an independent risk factor for stroke. Our study suggests that while diabetes contributes to the prediction of first-ever stroke risk in Chinese individuals, its impact may have been overestimated. Recent research indicates that the relative stroke risk associated with diabetes declines with age; elderly diabetics have only a 30% higher stroke risk compared to non-diabetics (Harris, 2023; Howard et al., 2023). This finding aligns with our logistic regression results (OR = 1.33, 95% CI 1.02–1.73, *P* = 0.033).

Dyslipidemia is also considered a risk factor for stroke. In our study, we excluded TC, TG, and LDL-C based on statistical analyses, incorporating HDL-C as the representative lipid variable in the stroke risk model. Two multi-province cohort studies in China found that HDL-C significantly enhances the predictability of stroke risk models (Wang et al., 2016; Xing et al., 2019). Our study found that HDL-C is a protective factor for first-time stroke, showing a negative linear correlation with stroke risk, the result consistent with previous research. For example, a meta-analysis included 25,678 patients with stroke revealed a linear relationship between HDL-C and stroke risk, with each 1 mmol/L increase in HDL-C reducing overall stroke risk by about 18% (Qie et al., 2021). Another observational study of 5,475 Chinese patients with ischemic stroke also showed a negative linear correlation, with each 0.3 mmol/L increase in HDL-C reducing ischemic stroke risk by about 7% (Sun et al., 2019). Additionally, a study of Black and White diabetic patients found a negative correlation between HDL-C and stroke, cerebral ischemia, and cerebral hemorrhage in type 2 diabetes patients (Shen et al., 2019). Mechanistically, HDL is known to provide multiple protective effects on blood vessels, including promoting cholesterol efflux from macrophages in arterial walls, enhancing endothelial function, and reducing inflammation and oxidative stress (Rosenson et al., 2013). While HDL-C is often considered a biomarker of HDL’s protective functions, high levels of HDL-C may actually impair HDL’s functionality. Consequently, some studies found nonlinear relationships between HDL-C and stroke risk, differing from our results. For instance, a large prospective cohort study showed a U-shaped relationship, with cumulative average HDL-C levels of ≤1.06 mmol/L or ≥2.05 mmol/L increasing stroke risk (Li et al., 2022a). A recent study also found that among individuals with high HDL-C levels (≥60 mg/dL), additional increases in HDL-C were associated with an increased risk of cardiovascular disease (Kim et al., 2023). Based on the consistency and differences with existing literature, our findings on HDL-C as a protective factor are reliable, but further research is needed to explore the U-shaped relationship between HDL-C and stroke risk in developing predictive models.

This study integrated the binary variable of “carotid atherosclerosis” into a first-stroke risk prediction model. The significant association between carotid atherosclerosis and first stroke (OR = 3.09, 95% CI 2.11-4.58, *P* < 0.001) validates its predictive efficacy. Previous studies have indicated that factors such as carotid artery plaque hemorrhage, the presence of lipid-rich necrotic cores within plaques, and the thinning or rupture of the plaque fibrous cap—as identified by magnetic resonance imaging—serve as predictive indicators for cerebrovascular events (Mono et al., 2012; Gupta et al., 2013; Saam et al., 2013). Additionally, the total plaque area provides predictive information regarding the risk of first ischemic stroke (Mathiesen et al., 2011). Carotid intima-media thickness has also been correlated with first stroke risk in hypertensive patients (Sun et al., 2020). By merging carotid intima-media thickening with atherosclerotic plaque formation into a single variable, this study simplifies the model but introduces potential instability.

### 4.5 Implications for theory development and clinical practice

Analyzing the formation of Fire and its role in mediating stroke from both TCM theory and modern medical perspectives is crucial. According to TCM theory, the process of disease development can be divided into two stages. In healthy individuals, as they age, Yin Jing gradually diminishes, and Yang Qi also weakens. As stated in Huangdi Neijing, “At the age of forty, Yin Qi is halved.” In other words, overall bodily functions decline, leading to Qi dysfunction. This can result in both Qi stagnation transforming into Fire and the formation of blood stasis and phlegm. The combination of phlegm and stasis can also lead to the formation of Fire. The Fire then promotes the transformation of blood stasis and phlegm into toxin (Liu et al., 2024), which damage blood vessels. This is a key stage that drives the progression from a high-risk state to stroke.

From a modern medical perspective, this process, spanning from the stage before the formation of Fire to the stage of Fire formation, can be associated with aging-induced mitochondrial dysfunction (Tyrrell et al., 2020), which results in abnormal energy metabolism and oxidative stress (Yin et al., 2016). This can lead to the burst of reactive oxygen species, lipid metabolism disorders, insulin resistance, and insufficient insulin secretion by pancreatic β-cells (Bhatti et al., 2017; Vamecq et al., 2012; Takano et al., 2023). Lipid accumulation and chronic hyperglycemia drive inflammatory responses, causing endothelial damage and abnormal blood flow, which results in the release of inflammatory mediators by damaged endothelial cells and activated platelets. In this subsequent stage, the Fire may represent chronic inflammation during atherosclerosis, perpetuated by positive feedback mechanisms that lead to progressive worsening of both inflammation and endothelial damage, ultimately resulting in plaque rupture.

On the basis of control of vascular risk factors, the use of herbs with the characteristics of homology of medicine and food to prevent the formation of Fire should be emphasized in clinical practice. Before the formation of Fire, especially in elderly populations with symptoms of Yin or Yang deficiency, the medicinal foods Cornus officinalis and Cistanche deserticola can be used. They not only alleviate deficiencies in Yin Jing and Yang Qi but also enhance mitochondrial function, and improve glucose and lipid metabolism (Fu et al., 2021). When phlegm and blood stasis symptoms manifest, the medicinal foods Citrus reticulata and Notoginseng can be employed to prevent the transformation of them into Fire. Research has demonstrated that these herbs, effective in resolving phlegm and promoting blood circulation, can also improve lipid metabolism disorders (Guo et al., 2024; Du et al., 2023). When the symptoms of Fire are prominent, the herb Gardenia jasminoides, renowned for its Fire-clearing properties, can be used. It demonstrates significant anti-inflammatory effects (Li et al., 2022b).

### 4.6 Limitations of the study

There are several limitations in this study. Firstly, the limited sample size presents an unavoidable limitation. Due to a high dropout rate, only 110 participants were included in the stroke group. The reduced number of participants may affect the predictive power of the models, especially the performance of the XGBoost algorithm, which benefits from larger datasets. Smaller sample sizes can lead to higher variance in model performance, making it more susceptible to overfitting. Overfitting occurs when a model learns the noise in the training data rather than the underlying patterns, resulting in poor generalization to new unseen data. Additionally, the limited sample size means that the model may not capture the full range of variability and complexity within the population, leading to potential biases. These biases can arise from underrepresentation of certain subgroups or specific characteristics, which can further compromise the model’s external validity. Secondly, the objectivity of syndrome differentiation (Bianzheng) needs improvement. In this study, the reliance on clinical physicians for syndrome differentiation could introduce bias. The heterogeneity within the same syndromes may limit the predictive power of the models. This intrinsic variability can make it challenging for models to identify clear patterns and relationships, ultimately reducing the generalizability of the results. Thirdly, this study was conducted in a Chinese population, so it is unclear if the findings can be generalized to other ethnic groups or regions. Differences in genetic makeup, environmental factors, healthcare practices, and cultural and dietary habits may affect the predictive accuracy of the models when applied to diverse populations. Therefore, caution is advised when using these models in different populations. Finally, the community screening data was limited, and thus lacked information on recently recognized stroke risk factors such as elevated lipoprotein(a) (Kumar et al., 2021), vitamin B12 deficiency (Yahn et al., 2021), and low urinary sodium excretion (Kieneker et al., 2018). This limitation may underestimate stroke risk in individuals heavily influenced by these factors, reducing the model’s predictive accuracy, especially in such populations. datasets.

### 4.7 Implications for future work

Firstly, future research should focus on increasing the sample size by reducing participant dropout rates and by recruiting a more diverse and representative sample. One effective strategy is to conduct regular follow-ups every 3 months instead of a single, long-term follow-up. This helps to maintain participant engagement and reduce dropout rates. Secondly, future research should use artificial intelligence diagnostic tools for data collection in TCM syndrome identification, such as face, tongue, and pulse diagnosis devices. Our team plans to use artificial intelligence devices to assist in collecting TCM information during community stroke screenings. Additionally, some research teams have developed TCM corpora based on clinical records (Zhang T. et al., 2020), providing standardized data for intelligent recognition of TCM syndromes. Deep learning techniques have also been applied to develope diagnostic prediction models for TCM syndrome differentiation (Chen et al., 2024). These emerging technologies will contribute to improving the objectivity of TCM syndrome differentiation. Thirdly, future research should prioritize rigorous external validation of these models to ensure their generalizability and reliability across diverse real-world clinical settings. This involves testing the models across different patient populations, healthcare environments, and TCM practitioners, thereby guaranteeing their applicability and trustworthiness in practical scenarios. Finally, future studies should validate the predictive performance of potential risk factors such as lipoprotein(a) and vitamins using more comprehensive databases like the National Health and Nutrition Examination Survey. If necessary, future efforts to develop a high-risk stroke database integrating traditional Chinese and Western medicine could incorporate these additional risk assessments. Incorporating such data would enhance the model’s predictive accuracy and interpretability.

## 5 Conclusion

High SBP, Fire syndrome, carotid atherosclerosis, advanced age, current smoking, and diabetes may be associated with an increased risk of new-onset stroke, while high HDL-C may serve as a protective factor. Strengthening the identification and management of the Fire syndrome in populations at high-risk of stroke may be a viable strategy to further reduce the incidence of new-onset stroke.

## Data Availability

The original contributions presented in the study are included in the article/Supplementary Material, further inquiries can be directed to the corresponding authors.
