# Supplementary material for: Predicting new-onset stroke with machine learning: development of a model integrating traditional Chinese and western medicine
Source: Front Pharmacol. 2025 Feb 21;16:1546878. doi: 10.3389/fphar.2025.1546878 (PMC11885310; doi:10.3389/fphar.2025.1546878)
Supplement: Supplementary file 1 [file Table1.docx]

Supplementary Material

# Supplementary Tables

**Supplementary Table 1** Basic characteristics of training set data before and after sampling.

| **Variables** | **Before sampling**  **(n=1248)** | **ROS**  **(n=2342)** | **RUS**  **(n=154)** | **SDG**  **(n=1248)** |
| --- | --- | --- | --- | --- |
| **Sex** |  |  |  |  |
| Male | 447 (35.8%) | 937 (40.0%) | 59 (38.3%) | 494 (39.6%) |
| Female | 801 (64.2%) | 1405 (60.0%) | 95 (61.7%) | 754 (60.4%) |
| **Age, Median [Q1, Q3], years** | 59.0 [53.0, 65.0] | 61.0 [54.0, 67.0] | 60.5 [54.0, 67.8] | 60.7 [53.9, 68.5] |
| **SBP, Median [Q1, Q3], mmHg** | 135 [122, 148] | 140 [130, 152] | 140 [130, 150] | 140 [127, 154] |
| **Diabetes** |  |  |  |  |
| Yes | 377 (30.2%) | 757 (32.3%) | 47 (30.5%) | 407 (32.6%) |
| No | 871 (69.8%) | 1585 (67.7%) | 107 (69.5%) | 841 (67.4%) |
| **HDL-C, Median [Q1, Q3], mmol/L** | 1.20 [1.00, 1.43] | 1.15 [0.970, 1.37] | 1.16 [0.993, 1.36] | 1.17 [0.954, 1.41] |
| **Carotid atherosclerosis** |  |  |  |  |
| Yes | 893 (71.6%) | 1896 (81.0%) | 128 (83.1%) | 1036 (83.0%) |
| No | 355 (28.4%) | 446 (19.0%) | 26 (16.9%) | 212 (17.0%) |
| **Current smoking** |  |  |  |  |
| Yes | 262 (21.0%) | 593 (25.3%) | 33 (21.4%) | 312 (25.0%) |
| No | 986 (79.0%) | 1749 (74.7%) | 121 (78.6%) | 936 (75.0%) |
| **Qi deficiency syndrome** |  |  |  |  |
| Yes | 605 (48.5%) | 1173 (50.1%) | 80 (51.9%) | 634 (50.8%) |
| No | 643 (51.5%) | 1169 (49.9%) | 74 (48.1%) | 614 (49.2%) |
| **Fire syndrome** |  |  |  |  |
| Yes | 642 (51.4%) | 1396 (59.6%) | 95 (61.7%) | 747 (59.9%) |
| No | 606 (48.6%) | 946 (40.4%) | 59 (38.3%) | 501 (40.1%) |
| **Yang deficiency syndrome** |  |  |  |  |
| Yes | 434 (34.8%) | 642 (27.4%) | 37 (24.0%) | 323 (25.9%) |
| No | 814 (65.2%) | 1700 (72.6%) | 117 (76.0%) | 925 (74.1%) |
| **Yin deficiency syndrome** |  |  |  |  |
| Yes | 406 (32.5%) | 890 (38.0%) | 58 (37.7%) | 477 (38.2%) |
| No | 842 (67.5%) | 1452 (62.0%) | 96 (62.3%) | 771 (61.8%) |
| **Phlegm-dampness syndrome** |  |  |  |  |
| Yes | 245 (19.6%) | 363 (15.5%) | 22 (14.3%) | 199 (15.9%) |
| No | 1003 (80.4%) | 1979 (84.5%) | 132 (85.7%) | 1049 (84.1%) |
| **Blood stasis syndrome** |  |  |  |  |
| Yes | 109 (8.7%) | 132 (5.6%) | 11 (7.1%) | 65 (5.2%) |
| No | 1139 (91.3%) | 2210 (94.4%) | 143 (92.9%) | 1183 (94.8%) |
| **New-onset stroke** |  |  |  |  |
| Yes | 77 (6.2%) | 1171 (50.0%) | 77 (50.0%) | 612 (49.0%) |
| No | 1171 (93.8%) | 1171 (50.0%) | 77 (50.0%) | 636 (51.0%) |

HDL-C, high-density lipoprotein cholesterol; LDL-C, low-density lipoprotein cholesterol; ROS, random over-sampling; RUS, random under-sampling; SDG, synthetic data generation; SBP, systolic blood pressure.

**Supplementary Table 2** The fitting model results of the AIC criterion using bidirectional stepwise regression method.

| **Variables** | ***Log-Odds*** | ***CI*** | ***P*** |
| --- | --- | --- | --- |
| (Intercept) | -4.61 | -6.05 – -3.19 | **< 0.001** |
| **Age** | 0.03 | 0.02 – 0.05 | **< 0.001** |
| **Systolic blood pressure** | 0.02 | 0.01 – 0.03 | **< 0.001** |
| **Diabetes** | 0.53 | 0.25 – 0.81 | **< 0.001** |
| **HDL-C** | -0.52 | -0.89 – -0.16 | **0.005** |
| **Carotid atherosclerosis** | 1.22 | 0.83 – 1.62 | **< 0.001** |
| **Current smoking** | 0.56 | 0.24 – 0.88 | **0.001** |
| **Qi deficiency syndrome** | -0.22 | -0.52 – 0.07 | 0.139 |
| **Yang deficiency syndrome** | -1.61 | -1.94 – -1.28 | **< 0.001** |
| **Phlegm-dampness syndrome** | -1.48 | -1.89 – -1.09 | **< 0.001** |
| **Blood stasis syndrome** | -1.82 | -2.52 – -1.16 | **< 0.001** |

HDL-C, high-density lipoprotein cholesterol.

**Supplementary Table 3** The fitting model results of the BIC criterion using bidirectional stepwise regression method.

| **Variables** | ***Log-Odds*** | ***CI*** | ***P*** |
| --- | --- | --- | --- |
| (Intercept) | -5.14 | -6.51 – -3.80 | **< 0.001** |
| **Age** | 0.04 | 0.02 – 0.05 | **< 0.001** |
| **Systolic blood pressure** | 0.02 | 0.01 – 0.03 | **< 0.001** |
| **Diabetes** | 0.50 | 0.23 – 0.78 | **< 0.001** |
| **HDL-C** | -0.52 | -0.89 – -0.16 | **0.005** |
| **Carotid atherosclerosis** | 1.23 | 0.84 – 1.63 | **< 0.001** |
| **Current smoking** | 0.64 | 0.33 – 0.96 | **< 0.001** |
| **Yang deficiency syndrome** | -1.51 | -1.83 – -1.20 | **< 0.001** |
| **Phlegm-dampness syndrome** | -1.38 | -1.76 – -1.01 | **< 0.001** |
| **Blood stasis syndrome** | -1.68 | -2.36 – -1.06 | **< 0.001** |

HDL-C, high-density lipoprotein cholesterol.

**Supplementary Table 4** Collinearity diagnosis of candidate predictive factors.

| **Variables** | **Collinearity statistic** | |
| --- | --- | --- |
|  | **Tolerance** | **VIF** |
| **Sex** | 0.64 | 1.563 |
| **Age** | 0.767 | 1.304 |
| **Systolic blood pressure** | 0.795 | 1.258 |
| **Antihypertensive treatment** | 0.797 | 1.255 |
| **Diabetes** | 0.974 | 1.026 |
| **HDL-C** | 0.896 | 1.116 |
| **Carotid atherosclerosis** | 0.798 | 1.253 |
| **Current smoking** | 0.659 | 1.517 |
| **Fire syndrome** | 0.168 | 5.936 |
| **Qi deficiency syndrome** | 0.17 | 5.883 |
| **Yang deficiency syndrome** | 0.177 | 5.641 |
| **Yin deficiency syndrome** | 0.184 | 5.434 |
| **Phlegm-dampness syndrome** | 0.242 | 4.132 |
| **Blood stasis syndrome** | 0.341 | 2.93 |

HDL-C, high-density lipoprotein cholesterol; VIF, variance inflation factor.
